# Supplementary material for: Using topic modeling to detect cellular crosstalk in scRNA-seq
Source: PLoS Comput Biol. 2022 Apr 8;18(4):e1009975. doi: 10.1371/journal.pcbi.1009975 (PMC9064087; doi:10.1371/journal.pcbi.1009975)
Supplement: S1 Table — Upper cutoffs for nFeatures has been set to relatively high values as we are interested in potential doublets. The percentage of mitochondrial genes (% mt) cutoff allows us to exclude dying cells. (PDF) [file pcbi.1009975.s001.pdf]

**Sample Name**	**features lower cutoff**	**features upper cutoff**	**% mt**
C51	500	3000	25
C52	500	2000	20
C100	500	6000	25
C141	500	7000	25
C142	500	6500	25
C144	500	4000	25
C143	500	6000	25
C145	500	4500	20
C146	500	4500	25
C148	500	7500	25
C149	500	7000	25
C152	500	6000	25
