# Supplementary material for: Using topic modeling to detect cellular crosstalk in scRNA-seq
Source: PLoS Comput Biol. 2022 Apr 8;18(4):e1009975. doi: 10.1371/journal.pcbi.1009975 (PMC9064087; doi:10.1371/journal.pcbi.1009975)
Supplement: S2 Table — We observe genes related to housekeeping and mitochondrial processes. These processes also exist in the reference population but, due to the low number of topics that we specified initially, it seems that they are only picked up in the second stage. Indeed, when 10 topics are used for the first stage, these genes appear at that stage (see Section PIC-seq dataset in the main manuscript and S3 Table). (PDF) [file pcbi.1009975.s002.pdf]

Topic ID	Genes	Notes
7	mt-Rnr2, mt-Co1, mt-Rnr1, mt-Nd5, mt-Nd1	appearing in over 1000 cells
8	H2-Aa,H2-Ab1, H2-Eb1, Fth1	appearing in over 800 cells
9	B2m, Eef1a1, Snord35nm Fth1	
