# Supplementary material for: Using topic modeling to detect cellular crosstalk in scRNA-seq
Source: PLoS Comput Biol. 2022 Apr 8;18(4):e1009975. doi: 10.1371/journal.pcbi.1009975 (PMC9064087; doi:10.1371/journal.pcbi.1009975)
Supplement: S3 Table — (PDF) [file pcbi.1009975.s003.pdf]

Topic ID	Genes	Notes
0	Fscn1, Calm1, Tmem123, Cd74, Malat1, Ftl1	Generally high expression in DCs, particularly 20h and 48h
1	Cst3, Ccl5, Cd74	DC specific genes, higher in 3h and some 20
2	mt-Rnr2, mt-Rnr1, mt-Cytb, mt-Nd4, mt-Nd1	Mitochondrial genes; not specific to a cell type topic
3	Igkc, Ighm, Igha, Gm42418, Gm26917, Jchain, Iglj1, B2m	Not specific to a cell type
4	Cdkna1a, Hspa5, Nfkbia, Ubc, Esd, Nr4a3	Similar expression across but some slightly higher in some T-cells
5	Snord32a, Ldha, Npm1, Ly6a, Eef2, Eef1a1, Trac	High in 48h T-cells
6	Ly6e, Trac, Stat1, Cd52, Gbp2	High in 3h T-cells
7	Gm42418, Gm26917, Hsp90ab1, Actb, Calr, Lars2, Myh9	Similar expression but slightly lower in some T-cells
8	Npm1, Ncl, Ldha, Ddx21, Nop58, Ccnd2	High in 20h T-cells
9	AC117232.5, Snord32a, Snord15b, Gm15710, Gm9794, Gm13456, Snord55, Rack1, Mir3091	High in 20h T-cells subset
