# Supplementary figures and images for: Using topic modeling to detect cellular crosstalk in scRNA-seq
Source: PLoS Comput Biol. 2022 Apr 8;18(4):e1009975. doi: 10.1371/journal.pcbi.1009975 (PMC9064087; doi:10.1371/journal.pcbi.1009975)

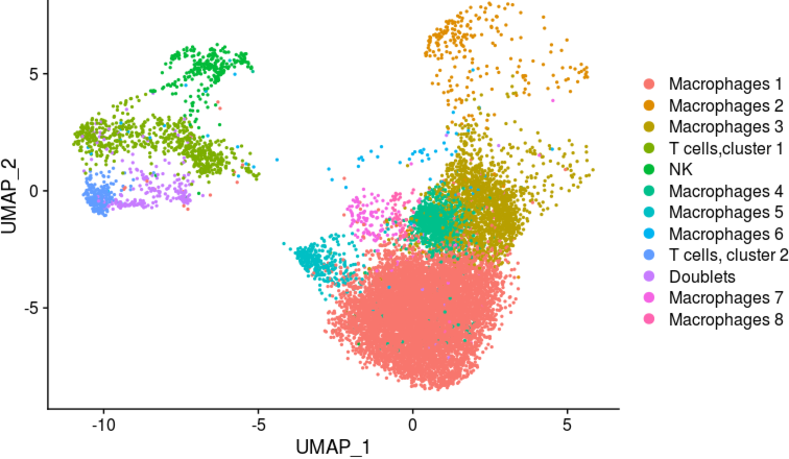

Supplement: S1 Fig — We have identified the cluster containing doublets based on expression of marker genes and annotation by DoubletFinder. (TIF) [file pcbi.1009975.s005.tif]

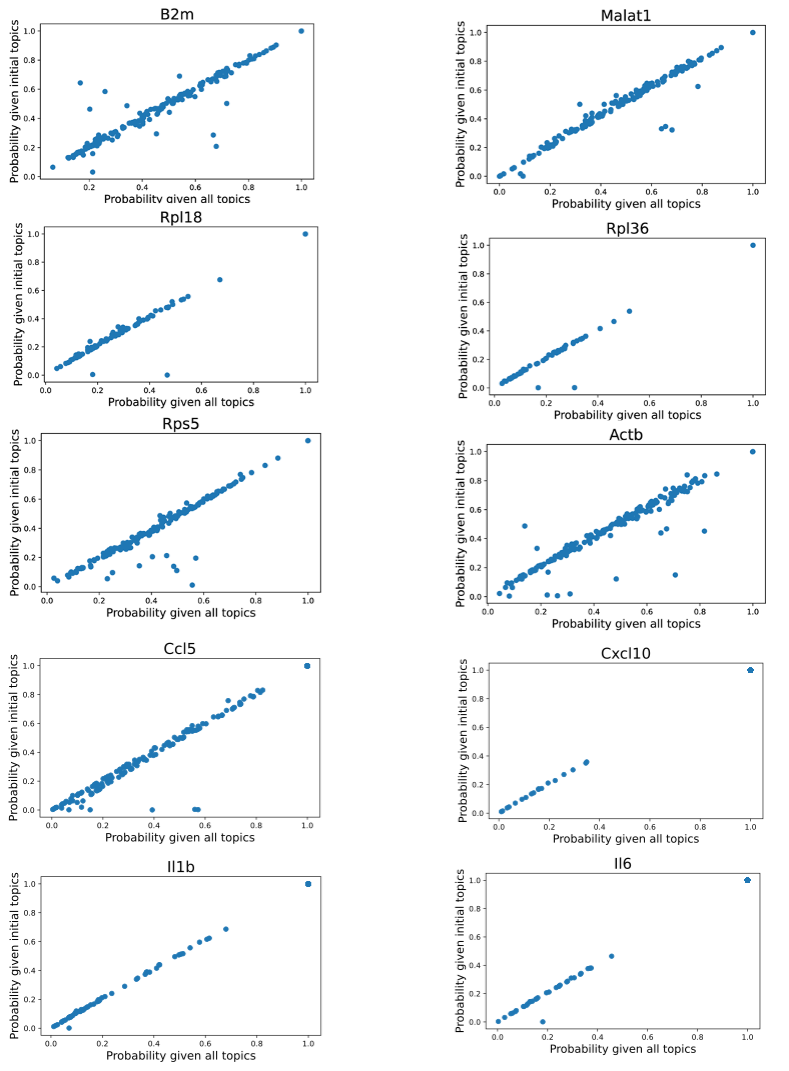

Supplement: S2 Fig — As expected, the probability of observing their counts is similar under the two models. (TIF) [file pcbi.1009975.s006.tif]

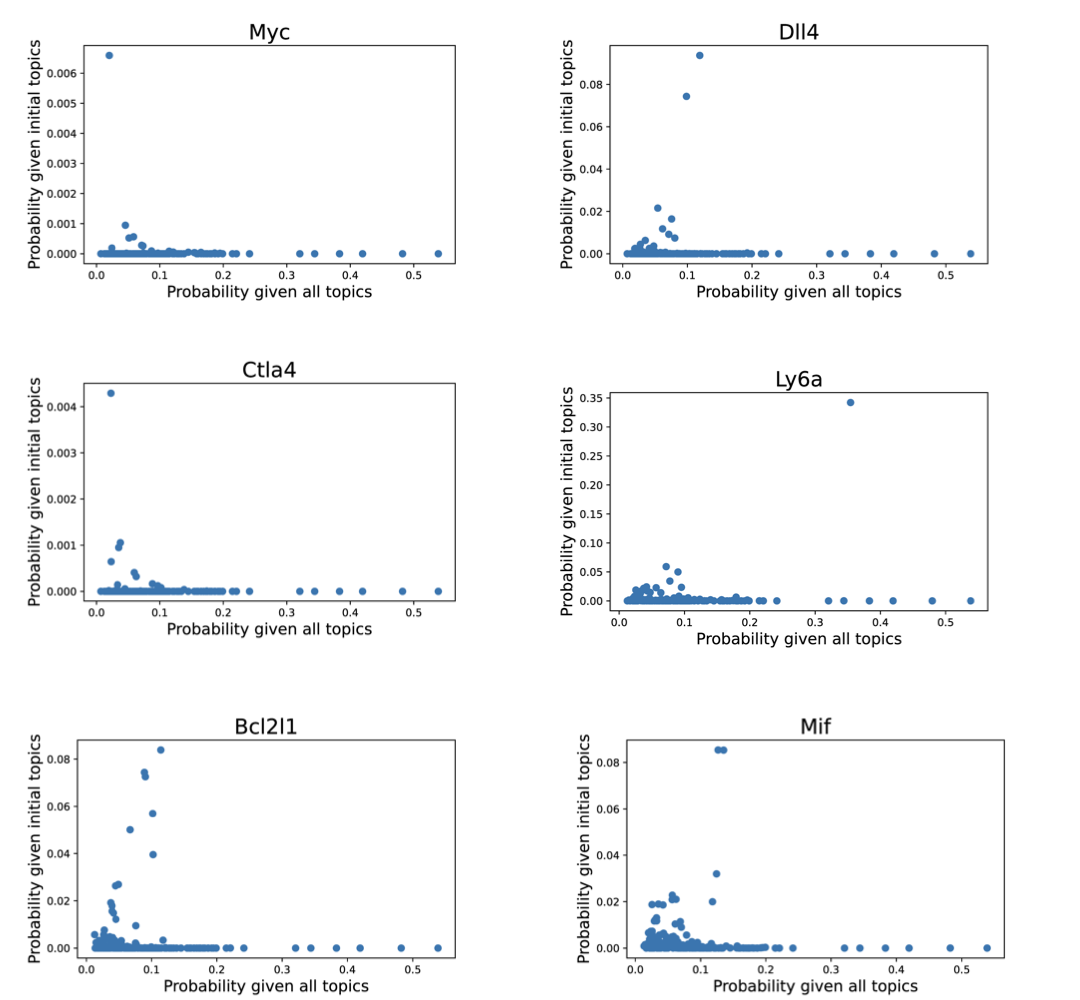

Supplement: S3 Fig — Their counts are observed with higher probability under the more complex model with new topics. (TIF) [file pcbi.1009975.s007.tif]

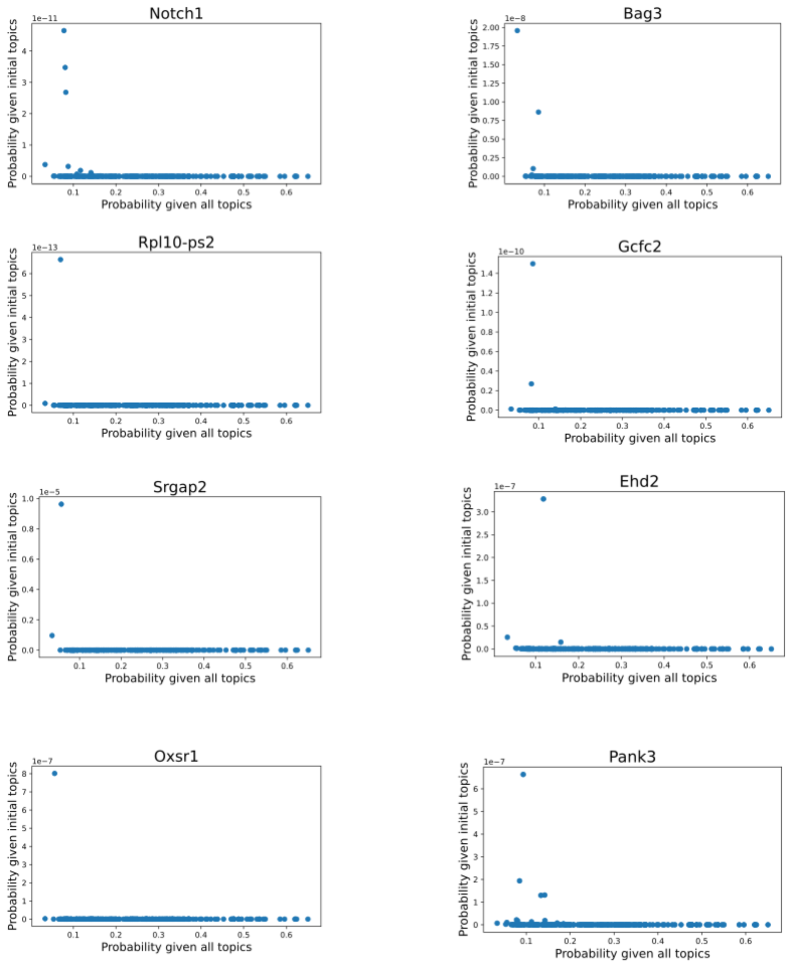

Supplement: S4 Fig — (TIF) [file pcbi.1009975.s008.tif]

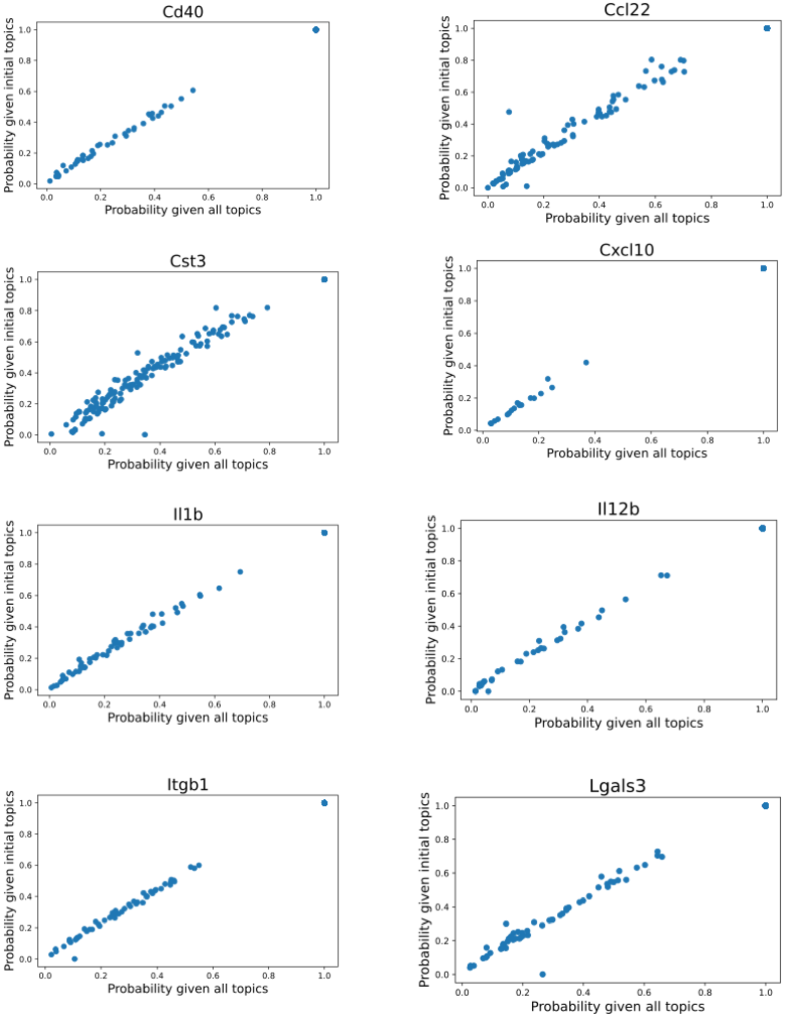

Supplement: S5 Fig — Similar probabilities can be observed under the two models. (TIF) [file pcbi.1009975.s009.tif]

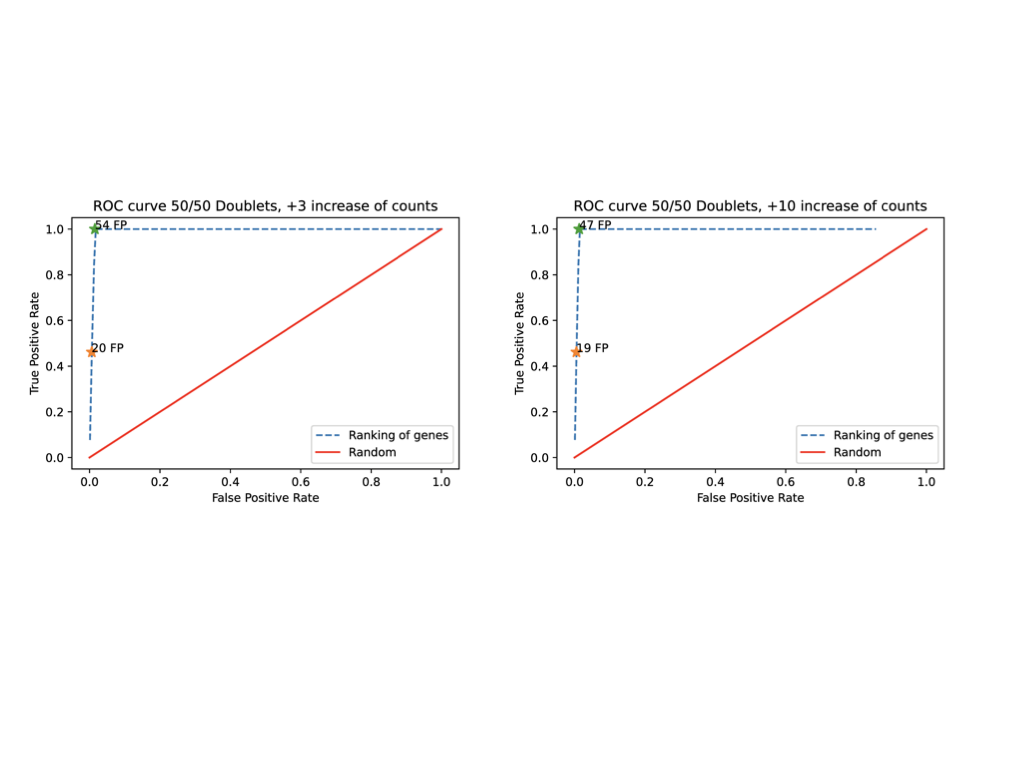

Supplement: S6 Fig — (TIF) [file pcbi.1009975.s010.tif]

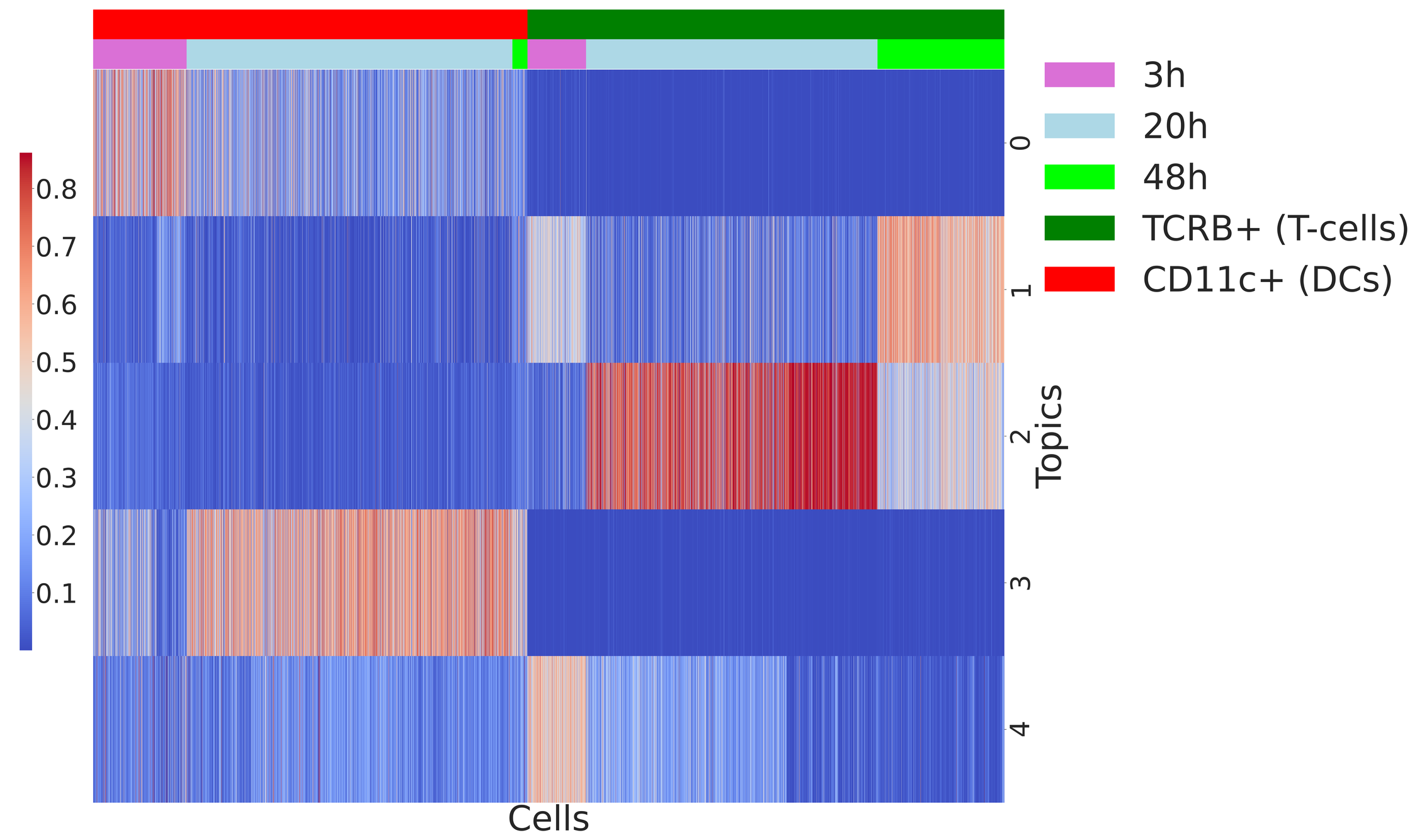

Supplement: S7 Fig — No topic is shared across all cells. We fit a model with 5 topics on the initial reference population, co-culture of DCs and co-culture of T-cells. As can be seen from the figure, we are capturing topics that are unique to DCs and T-cells. For example, topic 2, seems to be expressed in T-cells at 20h, while topic 3 is expressed in DCs at 20h. However, we would expect at least some genes to be expressed across both T-cells and DCs, for example housekeeping or mitochondrial ones, and these do not appear to be represented by any topics. It seems possible therefore that one result of under-specifying is that some processes that ought to be captured in stage 1 are actually captured in stage 2. (TIF) [file pcbi.1009975.s011.tif]

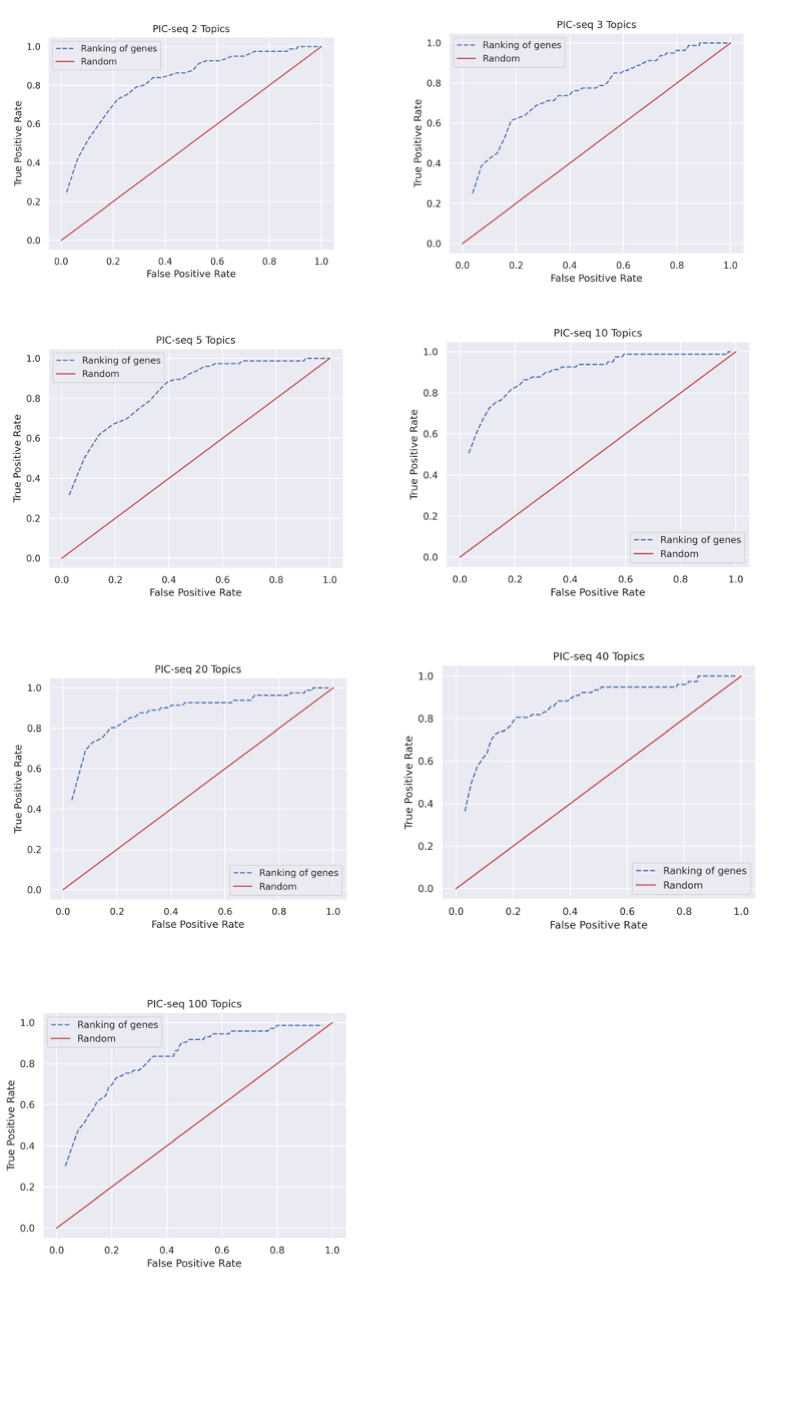

Supplement: S8 Fig — Ground truth is considered the genes identified by [10]. Following 10 topics, which can be considered the optimum for this dataset, the performance starts to drop and the ROC curves for 40 and 100 topics show decay. (TIF) [file pcbi.1009975.s012.tif]

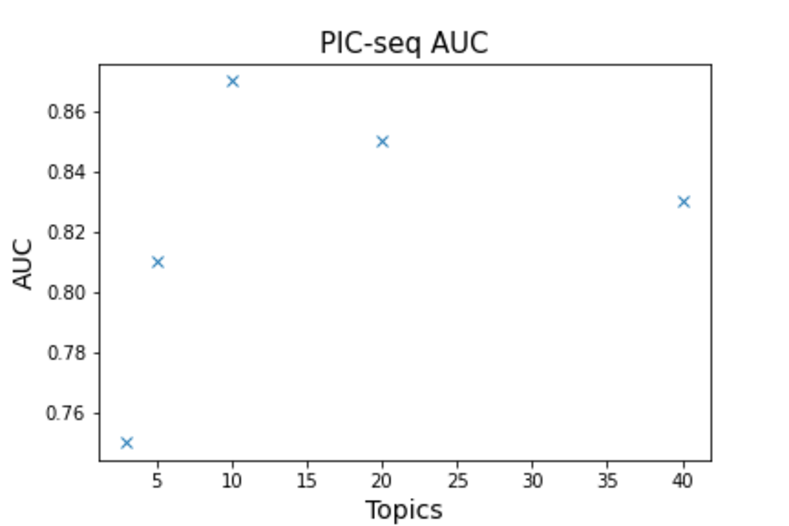

Supplement: S9 Fig — (TIF) [file pcbi.1009975.s013.tif]

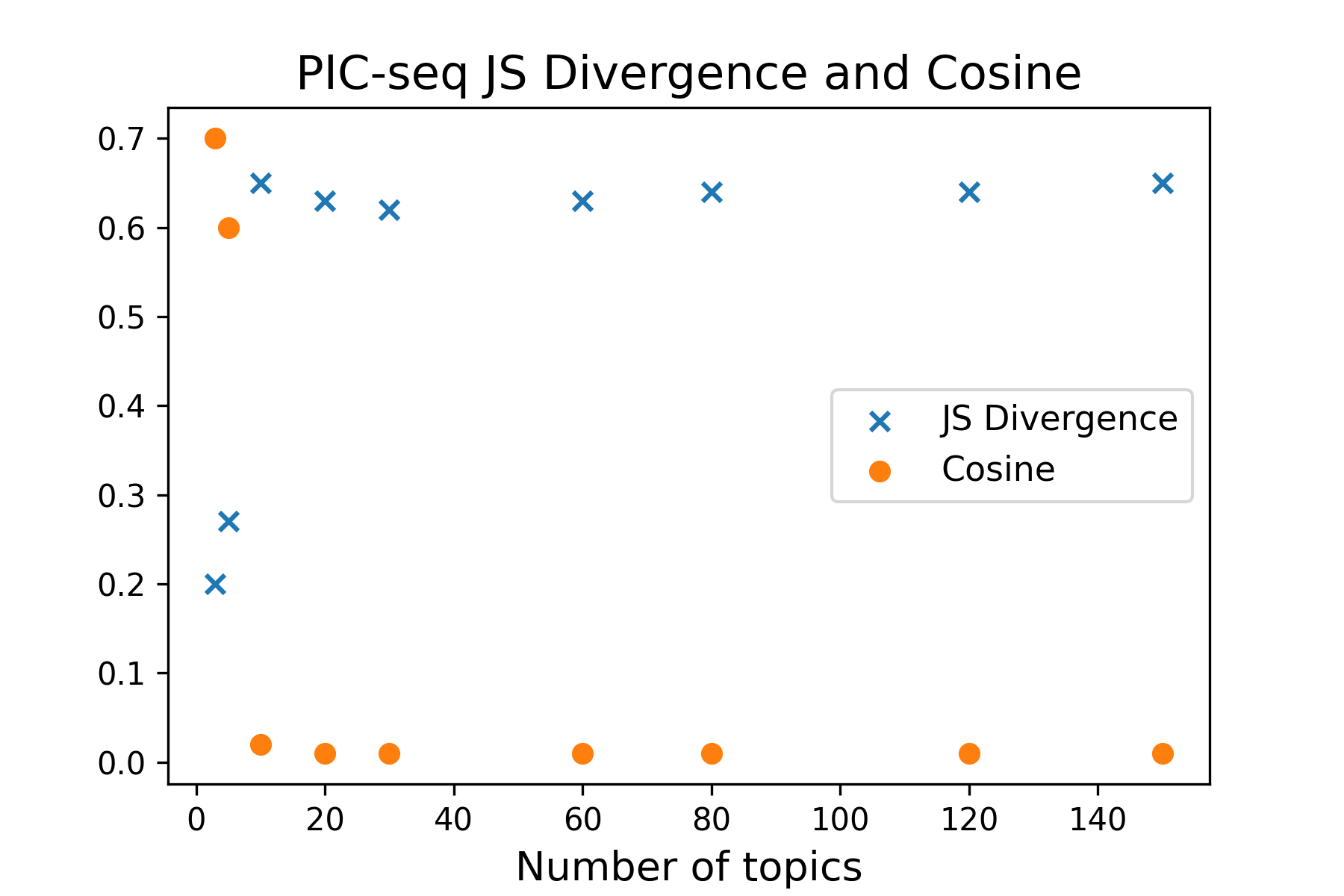

Supplement: S10 Fig — JS increases from 10 (higher is better) and cosine decreases from 10 (lower is better). (TIF) [file pcbi.1009975.s014.tif]

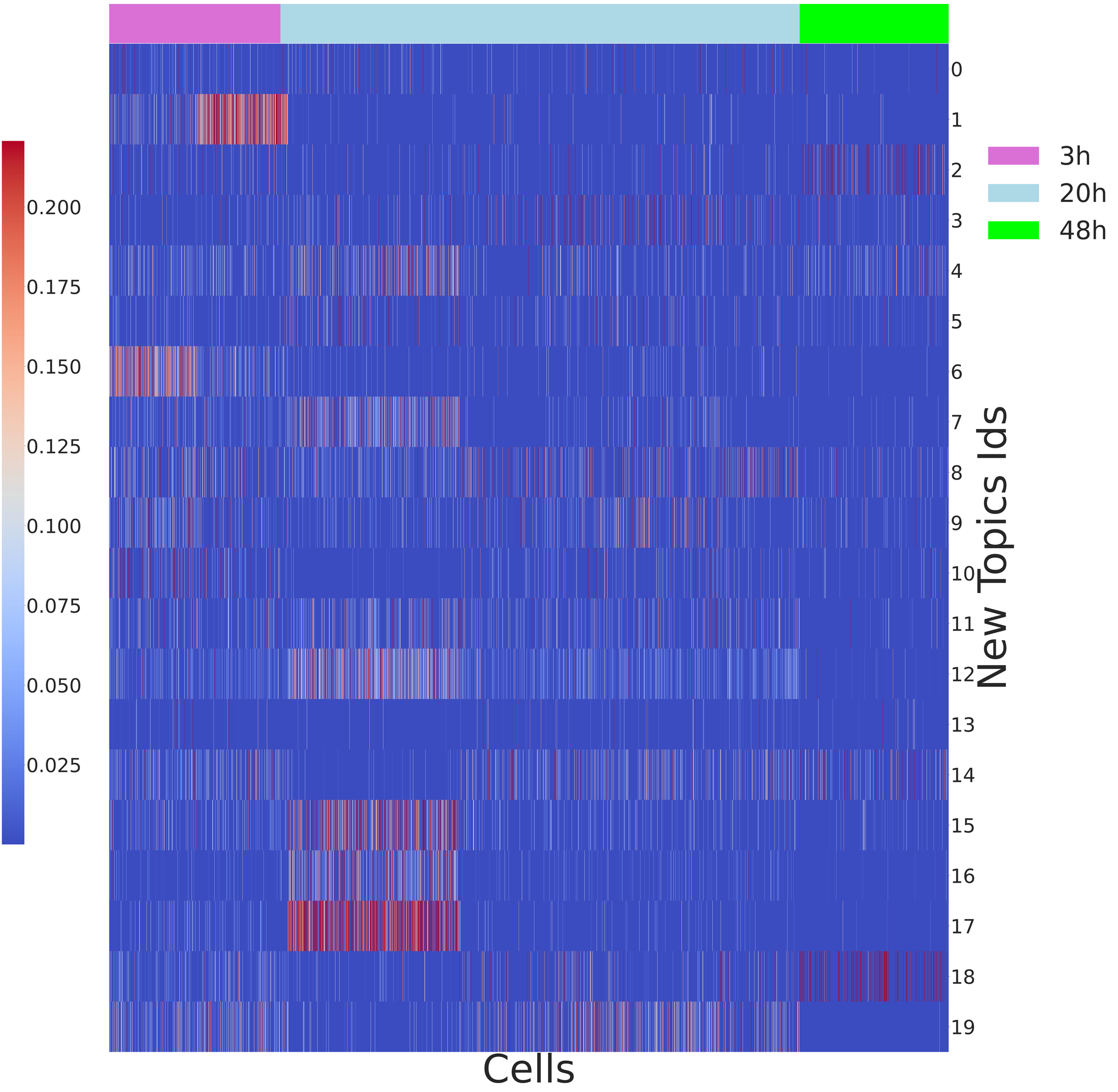

Supplement: S11 Fig — While some topics have higher probabilities associated with a timepoint, other topics appear expressed across all timepoints, so the gene expression shift is linked to the cell types that contribute to the PICs. (TIF) [file pcbi.1009975.s015.tif]
