# Supplementary material for: Using topic modeling to detect cellular crosstalk in scRNA-seq
Source: PLoS Comput Biol. 2022 Apr 8;18(4):e1009975. doi: 10.1371/journal.pcbi.1009975 (PMC9064087; doi:10.1371/journal.pcbi.1009975)
Supplement: Attachment — Submitted filename: response_to_reviewers.pdf [file pcbi.1009975.s016.pdf]

Dear Drs Nie and Ioshikhes,

Thank you for the opportunity to resubmit our manuscript titled "Using topic modeling to detect cellular crosstalk in scRNA-seq" to PLOS Computational Biology.

While the specific comments from the reviewers are addressed one by one in the next pages, we would like to summarise the main changes made to our manuscript.

Firstly, we have provided further information to ensure both the problem we are solving and the approach described are clear. More generally we have provided several clarifications to our approach and in order to facilitate the readers in their understanding we have added a figure of our 2-step LDA procedure.

We would like to highlight the fact that here we are proposing a novel method for identifying genes that change as a result of cell-cell interaction. Our approach relies on sequencing protocols that preserve the spatial proximity of cells such as PIC-seq. Specifically, we apply our methods to 2 datasets where the interacting populations are clearly labelled as such. We have included an application to 10x Chromium for completeness, as the question whether a population is made of technical doublets or interacting ones often arises in practice when analysing scRNA-seq data. Our results remain inconclusive due to the nature of 10x data and the fact that a reference population cannot be annotated with high confidence. However, considering the developments of lab based techniques for studying interactions, we believe our method would be applicable to future advances in the field.

Next, we have added further information about our synthetic experiments and additional plots can be found in the supplementary information.

We hope you now find the updated manuscript acceptable for publication in your journal. Please do not hesitate to contact us if you require any further information.

Best regards,
The authors

General note:
We would like to thank Reviewers #1 and #2 for their time and for providing us with invaluable feedback. The responses to each reviewer comment can be found annotated in blue below each point raised. Where appropriate we have added in quotes the specific textual changes to the manuscript and also provided the lines from the updated version of the manuscript.

Responses to Reviewers

Reviewer #1: Comments to the Author
In this manuscript, the authors seek to use LDA as part of a procedure to identify genes that change their expression as a result of cell-cell interaction. Code is made available through the first author's Github. I found the application to be relevant and current, and the method to be interesting. However, I did think that the exposition of the approach needed tightening, and had a few other minor queries/comments, as described below.

Minor points:

1. The LDA model (Section 2.1). Typo: "\alpha and \beta are Dirichlet priors" -- I think these are vectors.

This has now been corrected.

2. "Identifying topics linked to a cell type": for completeness and precision, it would be useful in this section to state the hypothesis that is being tested using Mann-Whitney's U.

There is now the following addition to this section: *"For each topic we group together cells from the same cell type as annotated in the reference and perform a Mann–Whitney U test on the topic-cell vectors. Under the null hypothesis, we assume a topic has the same probability in the two cell types"* (lines: 143-145).

3. In Table 1, why is 30 chosen as the lower bound for the number of topics? In general, how is an appropriate candidate set of values chosen?

We have changed the range of the number of topics we compute to include fewer than 30 topics and more than 100. Instead of setting for a specific value, we suggest a range is used as it can be seen from our results a range of topics can produce similar results in

terms of the computed metrics. We recommend users to consider the complexity of their dataset. For example, in the case of the PIC-seq data we have a complex dataset containing subtypes of T-cells and DCs as well as samples taken over different timepoints. Generally, underspecifying the number of topics might not allow to capture the complexity of the dataset. However, when there is a very high number of topics set for the model and they are not needed, they are not used and their probability is very low in the corresponding cells. Furthermore, as it can be seen from the supplementary figure 5 showing Jensen-Shannon divergence between a model with 10 topics and one with 150 topics, several of the topics from the more complex model correspond to a single topic from the model with 10 topics.

This has now also been clarified in the manuscript, lines 148-164:
“To select a suitable range for the number of topics, we compute average cosine distance, r, within topics as defined by [23] and pairwise Jensen-Shannon divergence as described in [24]. In the case of cosine distance a lower value indicates a more stable model while in the case of a Jensen-Shannon divergence higher values are preferred. For example, when trying to choose the number of topics for our PIC-seq dataset, we run LDA on the co-cultures several times with the following number of topics: 10, 20, 30, 60, 80, 120, 150. The table below shows the average cosine distance and the Jensen-Shannon divergence for a range of models. Since some of those models result in very similar values, we have investigated the effect of having a very large number of topics, 150. When it comes to future applications of our model, we recommend the expected complexity of the dataset is considered. We compute Jensen-Shannon divergence between topics in a model with 10 topics and a model with 150 topics. In supplementary information, S5 Fig. we show that several topics in the model with 150 topics are similar to the same topic in the model with fewer topics. We examine the contributions of topics to cells and we can see that in the case of a very high number of topics not all topics are used to model the expression..”

4. Some of the language needs a little tightening, e.g. "Ranking genes potential candidates of interaction", "Let N be the doublets/interacting population of interest" (is N the sample size?), missing articles (e.g. "We expect the probability of observing counts greater or equal to the actual count for gene changed...", "Metadata file was used to filter ...", "Full list of filtering cut-offs..."), avoiding contractions (e.g. "a gene we haven’t modified...", "It's important to note...")

Thank you for pointing these out. Our manuscript has now been carefully proofread to avoid missing articles and contractions, and we have made sure when using notation we explicitly clarify the meaning of any letters, e.g N.

5. A 2-stage approach is adopted here, in which LDA is first performed on a reference population, and then another LDA is performed to try to identify new topics that capture any

changes as result of interaction. I am not suggesting that the authors try this, but I wondered if they had instead considered a hierarchical model that would seek to model the two datasets jointly? If so, what would be the potential advantages/disadvantages of such an approach?

Modeling the datasets jointly would certainly be an interesting way of solving this problem. Such a model would introduce substantial additional complexity. We find the fact that we are capable of capturing genes that change as a result of interaction with our current approach strong motivation for investigating a more holistic model.

6. "Finding the number of topics" - here, average cosine distance is used, but are other options available? Why was this particular criterion chosen?

One of the inherent problems of topic models is choosing the number of topics. A popular approach for evaluating LDA is computing perplexity: the lower the perplexity, the better fit the model is for the data. Generally, perplexity drops when more topics are used. However, perplexity does not take into account topic interpretability.

Other metrics for measuring LDA performance have also been developed. Examples include average cosine distance and also adaptations of metrics generally used with probability distributions such as symmetric KL divergence and Jensen-Shannon divergence as presented by Cao, 2009, Arun, 2010 and Deveaud, 2014. Initially, we chose cosine distance as it is an easy to compute metric and it has been used successfully in practice to choose suitable LDA model parameters.

We have now added computed Jenson-Shannon divergence to the table as this is standardly used for probability distributions and similarly to the average cosine distance we get similar values for a range of topics. As previously discussed, if the number of topics is too low we risk not being able to capture the complexity of the data while if the number of topics is too high, topics are either not used to model the cells or multiple topics from one model correspond to one topic in a model with fewer topics (supplementary figure 5 showing Jensen-Shannon divergence heatmap of a model with 10 topics and a model with 150).

Reviewer #2: Pancheva et al. present a method for the analysis of cell-cell interactions through latent Dirichlet allocation (topic modeling). The methods introduced are interesting and I could see their potential impact when applied to current and emerging single-cell sequencing technologies. The paper in its current form is unclear in several places on the methods and the results presented, which limits comprehension of the main points being made. Specific concerns are listed below.

Major points

1. Definition of terms. I find the introduction of cell-cell communication via ligand-receptor interactions confusing, since ligands can in general diffuse, so might define a very different set of interacting cells than PICs. I urge the authors to be more careful / define more precisely what they mean by “an interaction”? In its current form, I don’t understand clearly when this refers to a general interaction between two cell types,. e.g. {T cell—Dendritic cell}, and when it refers to an interaction between/mediated by (the products of) two genes, e.g. cytokines/receptors/adhesion molecules, etc. This is an essential point as it is key to the paper. It also influences statements such as “do not allow for new interactions to be identified” (line 18) - cell/cell or gene/gene interactions?

Initial focus of understanding interaction from gene expression data has been on autocrine and paracrine communication as initial studies did not have access to physically interacting cells. As such, these analyses relied on ligand-receptor databases. Compared to standard scRNA-seq, PIC-seq allows for physically interacting cells to be analysed. However, it is worth noting that some of the genes the original PIC-seq paper (and we) identify to be changing as a result of interaction also include cytokines and chemokines receptors, for example, and as such there can be an overlap between some of the genes identified by the PIC-seq like approaches and ligand-receptor based databases. However, we do agree that standard ligand- receptor screening vs PIC-seq might capture different types of interactions between cells.

When discussing interactions, we mean cell-cell interactions. We do discuss costimulatory and adhesion molecules as they are directly linked to the presence of physically interacting cells.

We have made the following changes to the manuscript to clarify our point:

- *“One way to study interactions between cell types, widely used in scRNA-seq studies, relies on ligand-receptor pairs screenings”* (lines: 11-12)
- *“As these methods are based on databases of curated resources, they do not allow for new genes that change as a result of interaction between cell types to be identified…”* (lines: 19-20)

- *“The ability to capture PICs allows Giladi et al to study physical interactions between cells and potentially capture a novel set of genes that might be changing as a result of physical proximity”* (lines: 37-38)

We have also clarified that we are looking at physical interactions between cell types: lines 82 and 89.

2. Details on the two-step LDA procedure are not sufficient. Please explain the intuition behind this procedure in more detail (a diagram may help here).
In addition to clarifying the below mentioned points, we have also now provided a figure that will make it easier to understand the approach described in the manuscript. An overview figure of our approach can be found on page 5, Fig.1

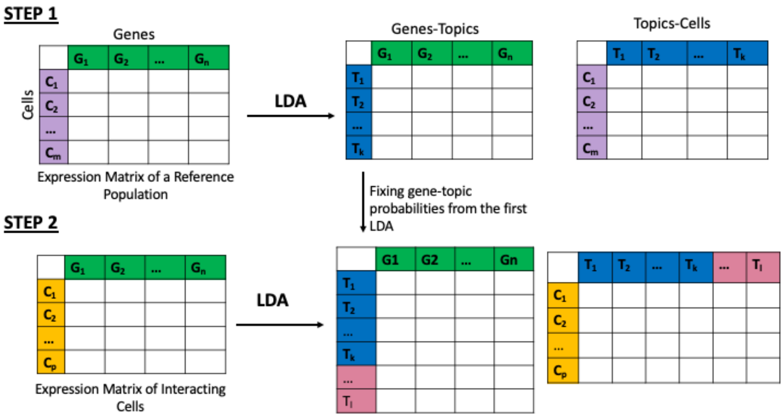

Specific questions:

is it reasonable to assume that cells are not interacting in reference population? Surely some could have been/were interacting by chance before they were dissociated and sequenced?

While we do agree that some cells might have interacted and separated, we would like to bring up the following two points:

1. In the case of the PIC-seq data: we fit the initial LDA on co-culture of T-cells and DCs (those are two separate co-cultures) and not on the monocultures that are also available. While we do recognise that there will be interactions, those would be between the same main cell type (within either T-cells or DCs populations) and we are more interested in interactions between different cell types (interactions between

T-cells and DCs). Furthermore, currently PIC-seq works only with mutually exclusive surface markers.

2. While it would be very interesting to see the profiles of cells that have interacted and then separated, these are data we do not have access to due to current ways of generating interaction datasets. However, even if such datasets become available where we have access to cells that have interacted and then separated, our method would also be applicable.

Use of the phrase "fixing topics" is not clear - as in: fixing the number of topics or fixing the entire distributions?

We apologise for the confusion. This has now been fixed to make our approach clearer and the text explicitly specifies that some of the topic-gene probability vectors in step 2 are fixed. In the manuscript this has been specified as follows:

- "*Fixing topic-gene probabilities learned from the reference*" ... line: 135
- Also in Fig 1 caption: "*We begin by fitting LDA on a reference population. Depending on the setting of the experiment the reference could be sorted cells (Boisset's dataset) or co-cultures of the cell types involved in the interaction (PIC-seq dataset). Using the topic-gene probability vectors we learn from this first LDA, we fit another LDA with some topic-gene probabilities fixed on the interacting population.*"

"By fitting LDA on the co-cultures of one cell type in a dataset..." does this really mean one cell type? Or one sample of cells? My understanding was that you are fitting LDA for the _set_ of cell types that are present in the entire (non-interacting) cell population sample? Please explain.

We apologise for the lack of clarity. We are fitting LDA on a co-culture of DCs and a co-culture of T-cells. To avoid the lack of clarity, we have now updated and the text where we describe the PIC-seq experiment now reads:

- "*co-cultures of the cell types in the PIC-seq dataset.*." (lines 123-124)
- "*By fitting LDA on the co-culture of T-cells and the co-culture of dendritic cells (DCs)...*" (lines 130-131)
- The figure caption of Fig 1 also now specifies: "*co-cultures of the cell types involved in the interaction*"

3. For the number of topics: the example of table 1 is not clear. The cosine distances at 30 topics and 100 topics are very similar, implying potentially high sensitivity of # topics

determined to the input data. Given the four distances calculated, it really begs the question: what are the cosine distances for topics < 30 and > 100?

A similar point has been raised by Reviewer 1. Please see our answers to points 3 and 6 raised by Reviewer 1.

4. “in the case of our synthetic experiments, we plot how choosing between 2 and 20 genes per topic affect the true positive and false positive rates” - where are these plots?

We apologise for the confusion. This text was revised since and we are plotting ROC curves now and not only showing true positives and false positives for values between 2 and 20 genes per topic. This sentence has been left over from a previous version of the manuscript and has now been removed.

5. Why is adding 10 counts to the expression of a gene a good model for evidence that its expression is influenced by interaction — What is justification for this? Does the absolute change in counts used affect the results?

Our synthetic experiments are designed on the basis that a physically interacting doublet is formed by different proportions of two other cells and by upregulating some genes. We agree that a count of 10 might seem too high and does not represent a typical count for a gene but rather a high value. To account for this, we have performed further experiments by upregulating the count by 1.5 and 3, those values were chosen as they represent values of gene expression with higher frequency in this dataset compared to 10. Taking as an example doublets created as an equal contribution of two cell types, we obtain the following number of false positives (FP) when we resolve the complete true set: 46 FP (increase of 1.5 to the counts), 54 FP (increase of 3 to the counts) and 42 FP (increase of 10 to the counts).

The ROCs in the synthetic experiments section are based on an increase of 1.5 while the ROCs for increase of 3 and 10 can be found in the supplementary material (only done for 50/50 doublets).

To account for these changes, the following text has been added to the manuscript:

- *“In order to obtain a ground truth for genes that change as a result of interaction, we modify the expression of some genes by adding 1.5, 3 and 10 to their total counts. Results are shown in Fig 2, Fig 3 and the supplementary information, S2 Fig, S3 Fig and S4 Fig.The value of 1.5 increase was chosen as it represents a typical count for a gene.”* (lines: 253-256)

More generally, more discussion of the results using synthetic data is needed. Figs 1 and 2 contain little information - what other plots would provide more detailed summaries of the inference results? e.g. (at least) plot more single genes than 2 in Fig 1;

We have now made the following changes to the manuscript:

- Included further plots of genes similar to the ones showing probabilities under the two models (Fig 2 in the updated manuscript) in the supplementary figures. Modified genes include: Myc, Dll4, Ctla4, Ly76a, Bcl2l1, Mif and genes we have not modified: B2m, Malat1, Rpl8, Rpl36, Actb, Rps5. Those are S2 Fig and S3 Fig on pages 17 and 18.

- Added experiments with different count number increase: Results can be seen on Figure 3 where now the ROCs with number of FPs include increase of count 1.5. The figures in the supplementary show the effect of increase of 3 and 10. Supplementary Figure 4.

in fig 2 would be more helpful to provide total # mis-classified genes since the absolute numbers are small (ROC curves not v informative).

We have now added the number of additional genes included in the ranking for our synthetic experiments to our ROC curves for the situations before the full truth set has been resolved.

Also: what is the effect of the abs count increase (if less than or greater than 10) on the results obtained?

Further experiments have been performed with 1.5 and 3 as those values represent more typical lower count values for a gene expression. Values larger than 10 have not been tried as 10 already represents an upper value for a gene count but different lower values have been tried and in both cases we are able to detect among the top ranking genes the ones that have been modified.

What is the detection limit? i.e. Can the method still pick up these interactions if they occur in 1 or 2 or 3 pairs of cells?

In one of our synthetic experiments, we modify the counts of 20 cells (out of 200) and our approach still manages to identify those genes in the ranking. As we are producing ranking of genes based on in how many cells, those genes require contribution from the new topics to have their counts modelled, we indeed can discover a gene that changes in very few cells and that would be reflected in the ranking of genes and if they wish a research scientist could explore genes that change in very few cells.

We have not done experiments with genes that change in 1 or 2 pairs of cells as those would be very rare interactions which might impact how easy it would be for them to be validated in a wet lab setting.

This has now been clarified in the synthetic experiments section, specifically the following text has been added:

- *"Additionally, we evaluate whether we can identify genes that change in a subset of cells by upregulating some genes in 10% of the total PICs population."* (lines: 283-284)
- *"We note that even if a gene is upregulated in as few as 20 cells that gene can still appear in the ranking, and we recommend also exploring genes which change in few cells when analysing results"* (lines: 287-289)

6. PIC-seq data: in Fig 3, it looks like there are some topics with very little variation across cells (e.g. topic 3), how to explain these? Also, I am curious what topics that pick up specific subsets of cells represent? e.g. topics 25 or 26. What are the top genes driving these? It would be helpful to include (supplementary) figures with genes contributing to topics for at least a good subset of the topics identified in Fig 3.

Samples were taken across different timepoints (3h, 20h, and 48h) and some of our topics do seem to capture the change of expression over time. Specifically, topic 25 has higher probability for 48h T-cells while topic 26 has higher probability for 3h DCs. This has now been added as a supplementary heatmap annotated with time (Supplementary Figure 6, page 20).

Topics with little variation across the cell types and timepoints contain housekeeping and mitochondrial genes or genes not showing high expression in T-cells and DCs (e.g Immunoglobin genes --Igkc, Ighm, Igha, etc.), specifically topics 3 and 29.

We have also added a table to the supplementary with a subset of topic id, genes and our interpretation of the topic, supplementary table 2, page 20.

Additionally to clarify the points mentioned above, the following text has been added to the manuscript:

- *"In addition to having high probability for a cell type, some of those topics have higher probability over the different timepoints. For example, topic 25 has high probability for T-cells during the 48h window. Similarly topic 26 is specific to DCs in the 3h time period, while topic 5 is DC specific for the 20h time period, see S6 Fig and S2 Table."* (lines: 302-305)

7. COVID-19 data: are the inconclusive results here due to: use of DoubletFinder + LDA not able to resolve interactions well (as the authors suggest), or due to the complexity/particularities of this dataset itself? Was DoubletFinder + LDA tried on any other dataset? In particular, there are a large number of clusters in the COVID dataset: perhaps trying DoubletFinder on a simpler (by # of cell types) 10X dataset would yield better results?

While in the manuscript we have described the application on our approach to a COVID dataset, we have also applied it to some internal (unpublished) data also generated by 10x that included DCs and several types of T cells. While those datasets were simpler compared to the COVID dataset in terms of number of clusters, the same problem persisted when it comes to results being inconclusive. As such, we consider the problem to be due to lack of clear labelling of reference population and previous research has shown that doublet tools' performance generally depends on a range of assumptions (in the case of DoubletFinder for example, that's doublet rate) and they do not achieve perfect sensitivity and specificity. The results of applying this method to a 10x dataset were included for completeness.

In the updated manuscript, we have added a clarification and a comment on tools for doublet detection which can be a potential explanation of why those results remain inconclusive.

- *"While we are capturing a shift in the expression of certain genes, our results are inconclusive, potentially due to the quality of our reference population as the reference is constructed based on the cells DoubletFinder annotated as singlets, and as such the reference might contain some interacting cells"* (lines: 402-406)
- *"While with DoubletFinder we have managed to label some of the doublets, computational tools for doublet detection do not achieve perfect sensitivity and specificity scores, so it is possible the reference population contains cells that exhibit signs of interaction and changes in the expression pattern of some potentially interesting genes."* (lines: 408-411)

Minor points

- line 399: While this is informative, the current setup of the 10x Chromium protocol is fully suitable for studying cellular crosstalk of physically interacting cells" - is this missing "not"?

In our updated manuscript, "*not*" has been added (line 431).
