# Supplementary material for: Using topic modeling to detect cellular crosstalk in scRNA-seq
Source: PLoS Comput Biol. 2022 Apr 8;18(4):e1009975. doi: 10.1371/journal.pcbi.1009975 (PMC9064087; doi:10.1371/journal.pcbi.1009975)
Supplement: Attachment — Submitted filename: response_to_reviewers.pdf [file pcbi.1009975.s017.pdf]

Dear Drs Nie and Ioshikhes,

Thank you for the opportunity to resubmit our manuscript titled "Using topic modeling to detect cellular crosstalk in scRNA-seq" to PLOS Computational Biology.

While the specific comments from the reviewers are addressed one by one in the next pages, we would like to summarise the main changes made to our manuscript.

As the main concern highlighted by the reviewers is related to the number of topics and practicalities of choosing the number of topics, we ran a perplexity analysis on a range of topics and proposed how to choose the number of topics. We also analysed how the number of topics affects the interacting genes identified by re-running our PIC-seq experiment with a different number of topics for the stage 1 LDA and compared how the ranked genes are affected. Furthermore, we updated the section of the manuscript titled “Choosing the number of topics” to provide an example and guidelines for how this should be done.

We have also added some further plots to the supplementary materials addressing questions about genes selected for our synthetic experiments.

We hope you now find the updated manuscript acceptable for publication in your journal. Please do not hesitate to contact us if you require any further information.

Best regards,
The authors

Responses to Reviewers

General note:
We would like to thank Reviewers #1 and #2 for their time and providing us with further feedback on the submitted manuscript. The responses to each reviewer have been annotated in blue below. We have also added specific quotes, tables, and figures from the manuscript where appropriate.

Reviewer #1: Thanks to the authors for largely addressing my points.

Two of the minor points I mentioned previously seem to have been missed:
1. The authors still assert that "α and β are Dirichlet priors that control..." -- but I think these are parameters of Dirichlet priors, not priors themselves.

Thank you for spotting this and we apologise for missing this in earlier versions of the manuscript. This has now been changed in the paper to “α and β are the parameters defining the Dirichlet priors over document-topic and topic-word multinomials”. (lines: 121-122)

2. Section title: "Ranking genes potential candidates of interaction" - I am not sure this currently makes sense. Is there a missing word?

Thank you for noting this. The section title has now been changed to “Ranking genes as potential candidates of interaction”.

Reviewer #2: In this revision the authors have addressed many of the concerns and the manuscript is improved for it. However, important questions remain, these are listed below.

1. Regarding the choice of number of topics, I understand that this is in general a challenge that perhaps no single metric can adequately quantify, but I still find the discussion in light of the metrics used (cosine distance and Jensen-Shannon divergence) to be inconclusive/not informative enough practically for potential users. For both these metrics the differences between a small and large number of topics is minimal. I am not sure what the observation of Fig. S5 adds? Is it not entirely expected that some of the topics from a many-topic model be contained (to some extent) in the topics of a few-topic model?

Another question is: why did the average cosine differences in Table 1 change between the initial submission to the revision?

Thank you for highlighting this. A more detailed discussion on how we addressed the identified issues about choosing the number of topics, can be seen below, including results from additional experiments.

The previously used S5 Fig has been removed as we did not feel it added to the contribution of the paper.

We apologise for any confusion the differences in cosine distance might have caused. The reason why the average cosine distance changed between the original submission and the revision was due to an error in the original manuscript, in which we presented results derived from out of date values of the hyperparameters.The correction was done in the first revision of the manuscript and results were consistent in that version.

Details on choice of the number of topics can be found in response to the next point. Furthermore, we have done additional experiments that suggest perplexity is more useful in this application for choosing the number of topics. We have removed the cosine similarity and JS divergence score and replaced that paragraph with perplexity analysis. A list of the changes made to the text can be seen in answer to your question below.

2. Practically, my concern is that there are no clear guidelines on choosing the number of topics for a user. Judging on cosine dist or JS, one would choose low (10) or low/high (10/150) numbers, respectively. Yet in examples, e.g. PIC-seq dataset, 30 topics are used. This may be due to the taking into account of “complexity” (which is not to my understanding well defined). For this method to be useful and adopted, this must be clarified. I think it will also be important to see some comparison of results with different numbers of topics, e.g. how do the heatmaps look for the PIC-seq dataset with 10 or 50 or 100 topics? These could be included as SI figs.

We agree that having some practical guidelines for determining the number of topics would make the technique more usable. To that end we have performed several additional experiments with the following overall conclusions (more details below):

1. We have assessed three LDA metrics (perplexity, cosine, and JS) across a wide range of values for the number of topics in Stage 1. All three plateau as K is increased beyond ~10 for the PIC-seq dataset. This suggests that a value of around

10 is appropriate. The perplexity plot is now included in the paper (and below) and the cosine and JS plots are provided in the supplementary material.

2. The goal of the proposed approach is recovering interacting genes. In addition to computing the measures described above, we have also computed the AUC for this task as the number of topics in stage 1 LDA increases. We find that this change is highly correlated with perplexity / JS / cosine: the AUC increases until K = 10 and then decreases (more gradually) as K is increased further.
3. This suggests that a sensible practical approach to determining the number of topics is to increase K until perplexity levels off (or cosine, or JS) and use this value of K for analysis. Our results also suggest that it is better to err on the side of over-specifying (higher K) than under-specifying (lower K).
4. In the light of the new experiments, we have redone our PIC-seq analysis using 10 topics as this has been identified as an optimum value.

The results of this extra analysis have been summarised in Section “Choosing Number of Topics” , and more details are provided in the Supplementary S8Fig, S9Fig, and S10Fig.

The text of this section now reads (lines: 147-161)

Choosing number of topics

To select a suitable range of topics, we compute perplexity for a range of topics starting with 2. A lower perplexity score is an indication of a better model. We note that perplexity decreases rapidly up to K = 10 and then flattens out. We also measure cosine and JS which show very similar patterns (Supplementary S10 Fig). Such behaviour is common when fitting LDA-style models and it is typical to choose the smallest value of K that is able to explain the data: i.e. the value of K at the point in which the perplexity flattens out. In Fig. 2, for example 10 would be a suitable number of topics for that dataset. Our goal is to recover interacting genes. To demonstrate that this strategy for choosing the number of topics is appropriate for that final goal, we show the effect the number of topics has on the Area Under the ROC curve. A plot of number of topics versus AUC can be seen in S9 Fig and shows agreement with the perplexity plot: optimal results are observed for K = 10, the value at which the perplexity flattens out. While performance is relatively consistent, the ROC curves with higher number of topics show some decay.

Perplexity Analysis

The plot shows the perplexity starting with 2 topics for the stage 1 LDA. As it can be seen from the plot, the perplexity drops till about 10 topics are reached and then stabilises with some fluctuations. Thus, 10 topics is a good value for the first LDA. This has now been added as Fig. 2. Those perplexity values follow a similar trend to JS divergence .

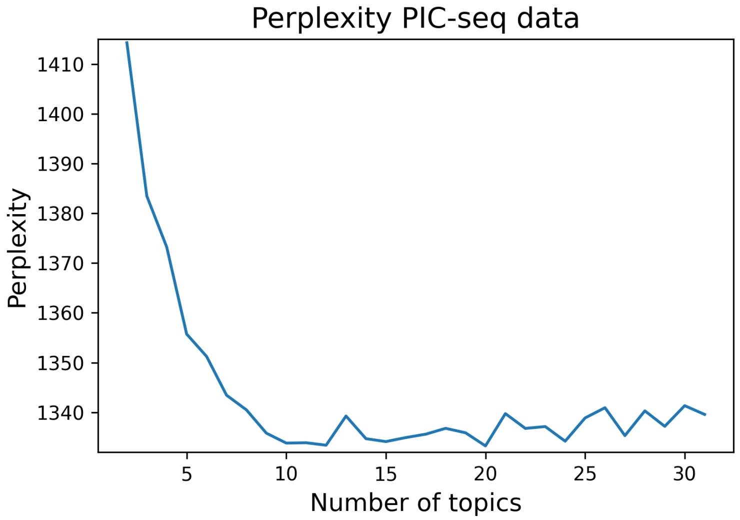

(NEW) Fig 2: Illustrating the drop of the perplexity as the number of topics increases. In this particular case, a suitable number of topics would be 10. After that as the number of topics increases, the perplexity values start to fluctuate.

Number of topics and effect on ranked genes

To evaluate the effect on the number of genes related to interaction discovered under different models, we plot ROC curves for different number of topics for stage 1 LDA.

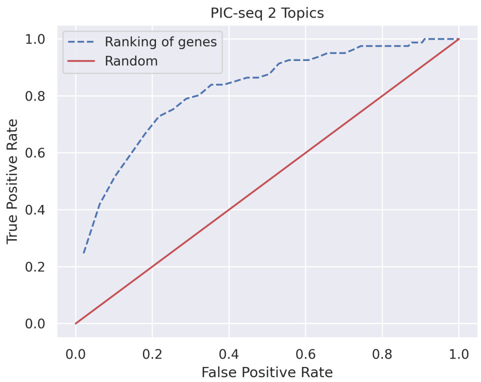

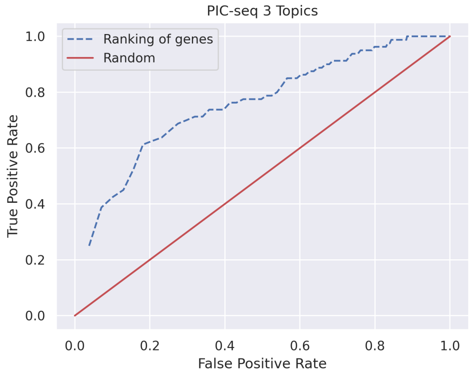

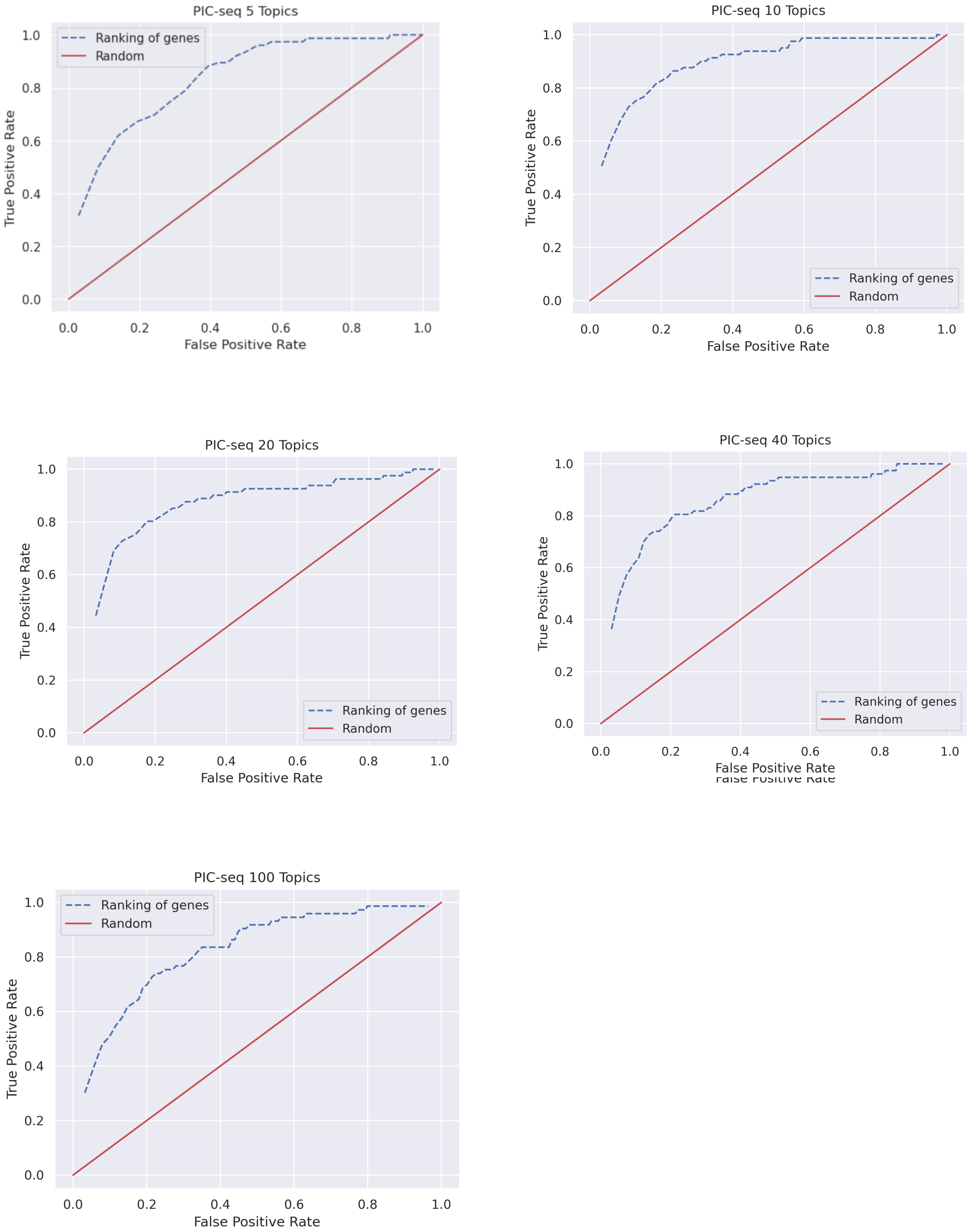

(NEW) S8 Fig: ROC curves for a range of topics. Ground truth is considered the genes identified by Giladi et al (81). Following 10 topics, which can be considered the optimum for this dataset, the performance starts to drop and the ROC curves for 40 and 100 topics show decay.

These plots are also summarised via plotting the number of topics versus AUC:

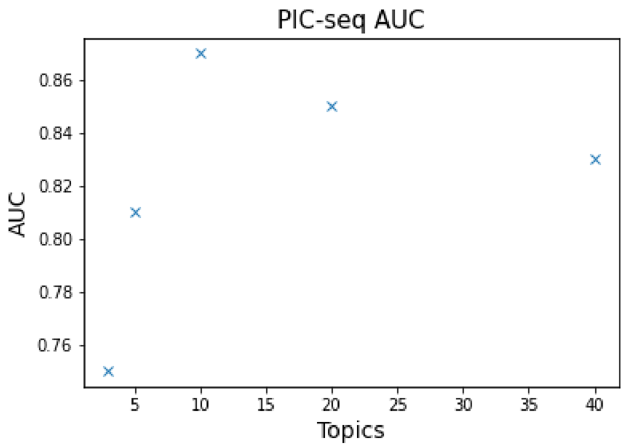

(New) S9 Fig: Area under the curve (AUC) is high for the optimal value of topics (10) and decays slowly after.

As can be seen from the ROC curves and AUC scores for a range of topics, optimum performance is reached when 10 topics are used as shown by the perplexity plot originally. AUC gradually decreases as K increases beyond 20. This plot is S9 Figure in the Supplementary.

Although there is no simple rule for specifying K, we believe that these additional results provide a sensible starting point – the number of topics at which point the perplexity stops decreasing.

As seen above, performance degrades more quickly when the number of topics is under-specified than it does when the number of topics is over-specified. We have investigated this, and documented our findings in S7, S8, and S9 of the supplementary document.

3. For new Fig 2, I appreciate the plots of additional genes in SI. However, the specific genes used are concerning, since the unmodified genes are almost all unrelated to immune cell phenotypes, i.e. characterizing general (housekeeping) cell processes (ribosome, actin). I think it is important to assess this in the analysis: switch the gene sets, and modify the expression of those in Fig S2, leaving the genes from Fig S3 unmodified, how would this affect the predictions of LDA?

While the initial synthetic experiment of modified genes included immune genes, the results are not dependent on the particular random genes chosen. To address the specific concern of only showing unmodified housekeeping genes, we have also added immune related

genes to our initial plots. Additionally, we have performed a second synthetic experiment where we randomly sample genes we modify the expression of.

To address this point:

1. Please find below and in the supplementary some other example plots of genes we have not modified that are immune related from the previously discussed synthetic experiments. Those plots have been added to Supplementary figure 2.

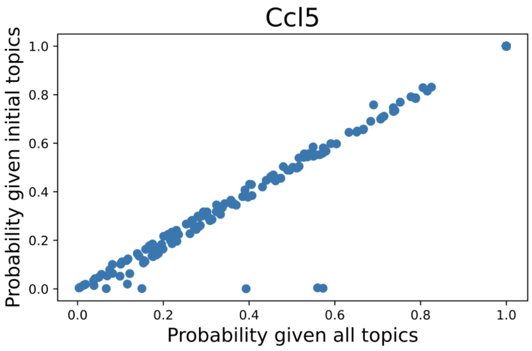

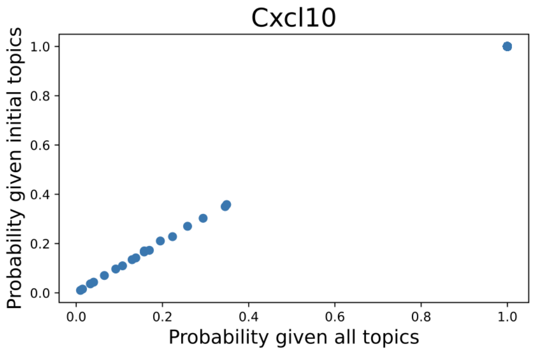

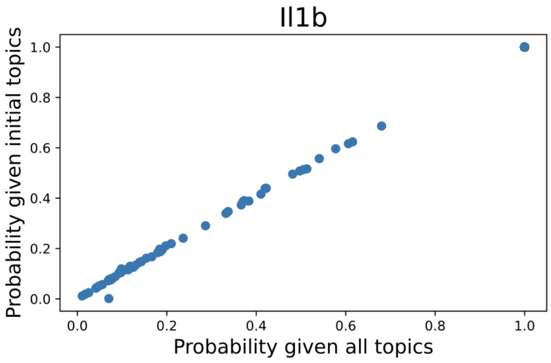

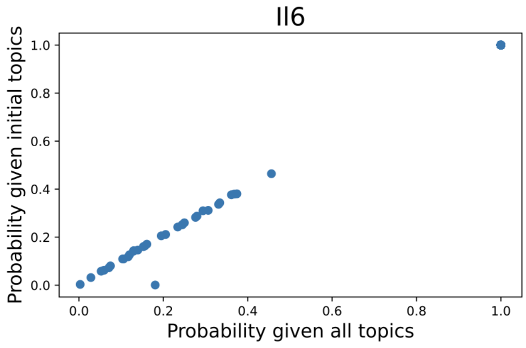

2. We have performed the suggested experiment where we randomly sample genes from the full list of genes using the random function in Python and modify their expression as previously explained. Similarly to the previously described experiments, we plot genes we have upregulated and examples of unmodified unmodified genes. The random gene set of modified genes consists of: 'Gcfc2', 'Wdsub1', 'AU040320', 'Pank3', 'Dcaf12', 'Gm26669', 'Ehd2', 'Bag3', 'Rpl10-ps2', 'Notch1', 'Ppm1g', 'Oxsr1', 'Nrarp', 'Ppp3ca', 'Rpl28-ps1', 'Stbd1', 'Srgap2', 'Cpped1', 'Gm10420', 'St6galnac3'.

Plotting the probability given initial topics vs probability given all topics of the modified genes above; those are genes we have changed the expression of. A subset of those genes are also included in the paper as Supplementary Figure 4.

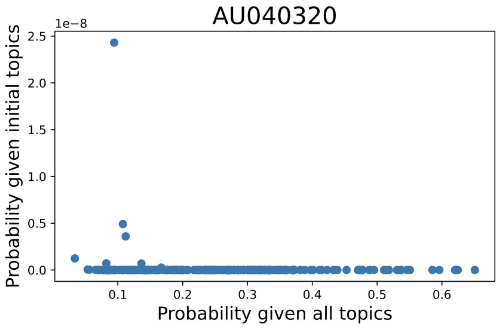
AU040320
1e−8
Probability given initial topics
Probability given all topics

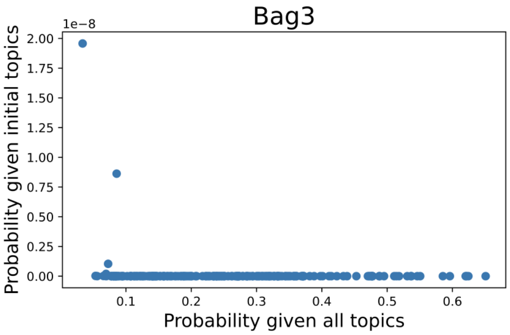
Bag3
1e−8
Probability given initial topics
Probability given all topics

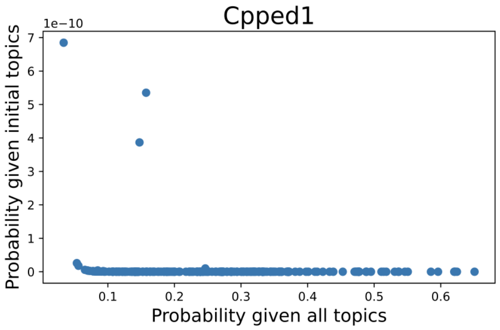
Cpped1
1e−10
Probability given initial topics
Probability given all topics

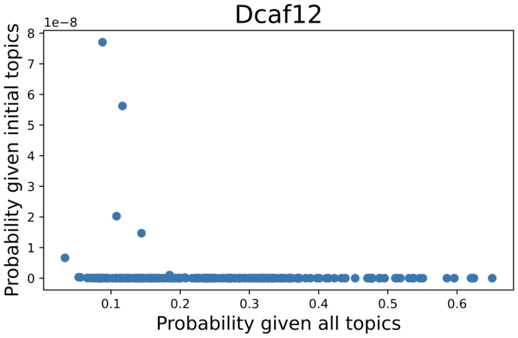
Dcaf12
1e−8
Probability given initial topics
Probability given all topics

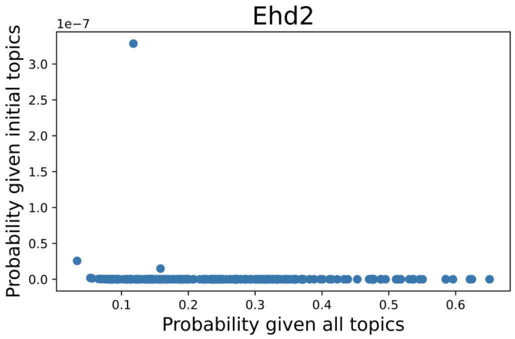
Ehd2
1e−7
Probability given initial topics
Probability given all topics

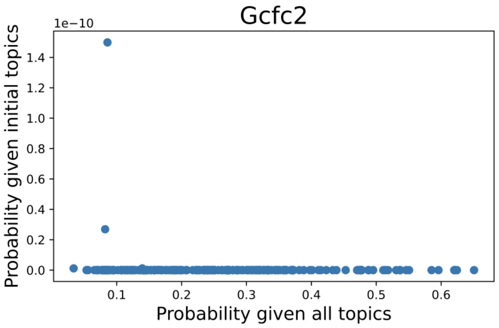
Gcfc2
1e−10
Probability given initial topics
Probability given all topics

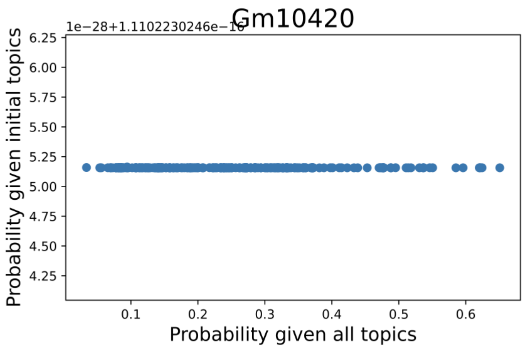
Gm10420
1e−28+1.1102230246e−16
Probability given initial topics
Probability given all topics

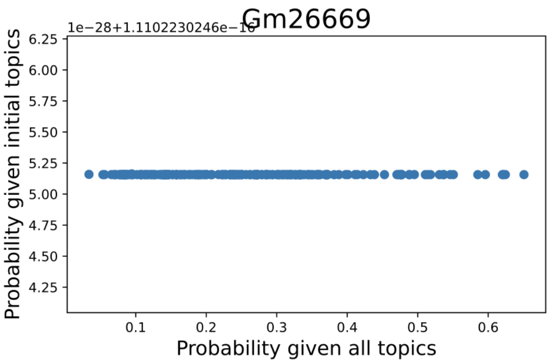
Gm26669
1e−28+1.1102230246e−16
Probability given initial topics
Probability given all topics

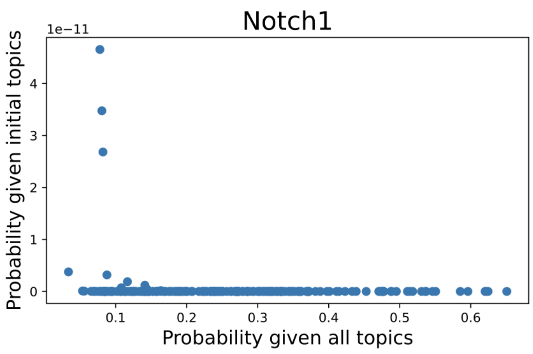
Notch1
1e−11
Probability given initial topics
Probability given all topics
0 1 2 3 4
0.1 0.2 0.3 0.4 0.5 0.6

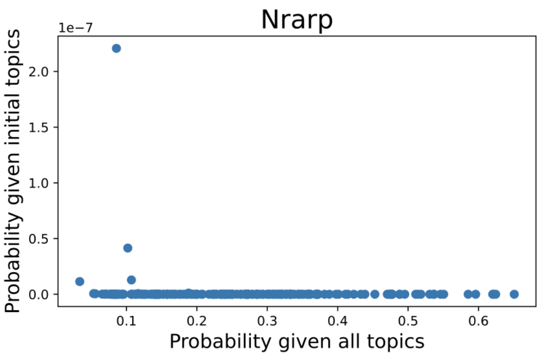
Nrarp
1e−7
Probability given initial topics
Probability given all topics
0.0 0.5 1.0 1.5 2.0
0.1 0.2 0.3 0.4 0.5 0.6

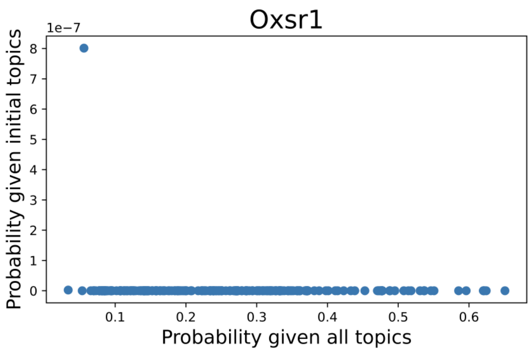
Oxsr1
1e−7
Probability given initial topics
Probability given all topics
0 1 2 3 4 5 6 7 8
0.1 0.2 0.3 0.4 0.5 0.6

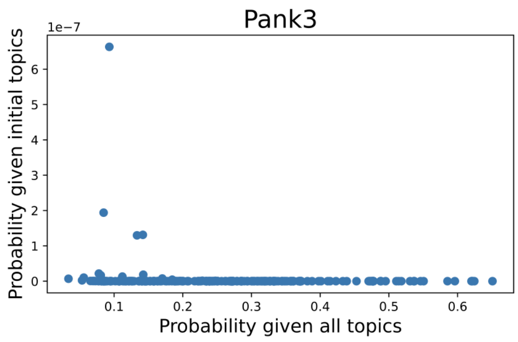
Pank3
1e−7
Probability given initial topics
Probability given all topics
0 1 2 3 4 5 6
0.1 0.2 0.3 0.4 0.5 0.6

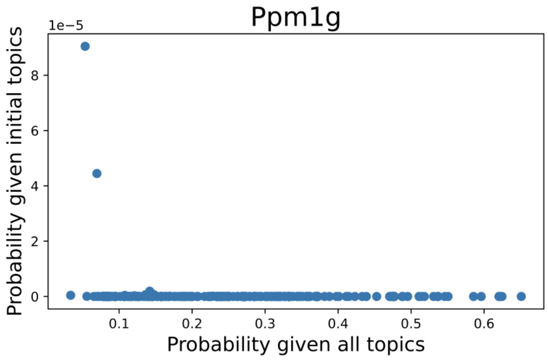
Ppm1g
1e−5
Probability given initial topics
Probability given all topics
0 2 4 6 8
0.1 0.2 0.3 0.4 0.5 0.6

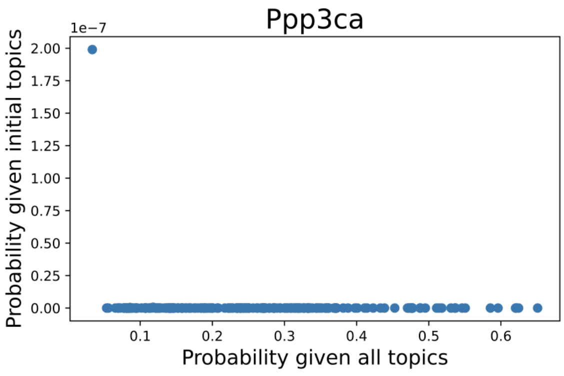
Ppp3ca
1e−7
Probability given initial topics
Probability given all topics
0.00 0.25 0.50 0.75 1.00 1.25 1.50 1.75 2.00
0.1 0.2 0.3 0.4 0.5 0.6

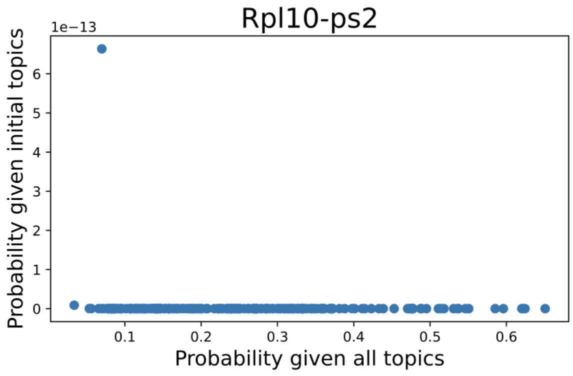
Rpl10-ps2
1e−13
Probability given initial topics
Probability given all topics
0 1 2 3 4 5 6
0.1 0.2 0.3 0.4 0.5 0.6

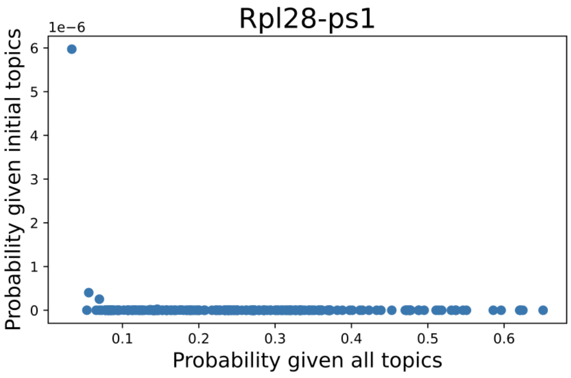
Rpl28-ps1
1e−6
Probability given initial topics
Probability given all topics
0 1 2 3 4 5 6
0.1 0.2 0.3 0.4 0.5 0.6

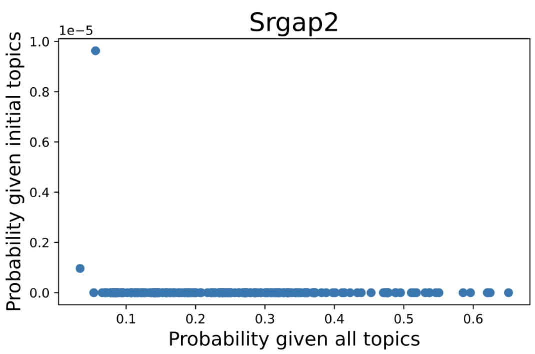

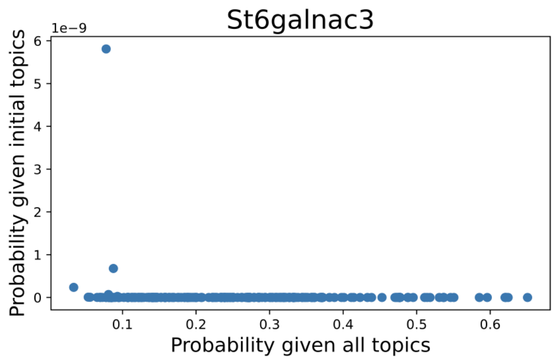

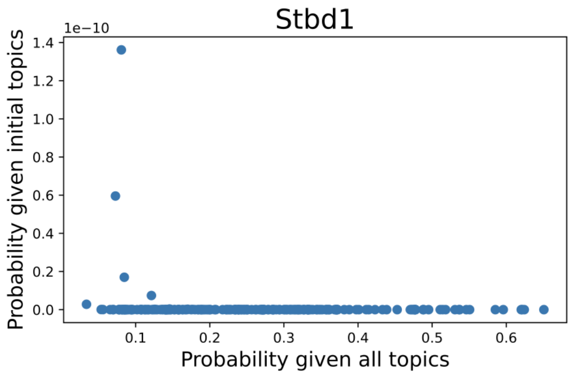

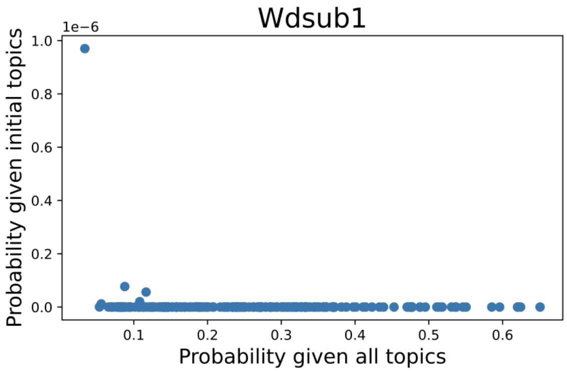

Here are genes we haven't modified the expression of. We choose to plot some genes generally related to immunity. This has been added to the manuscript as Supplementary Figure 5 with the following text "Additional synthetic experiment showing genes with unmodified expression. Similar probabilities can be observed under the two models."

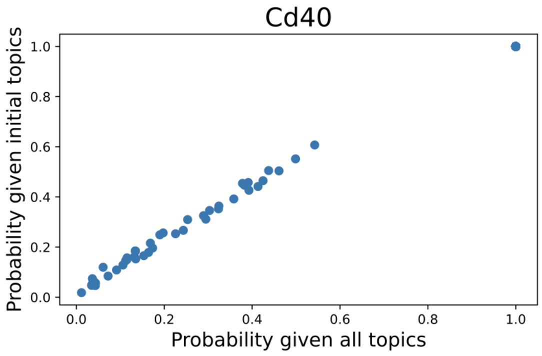

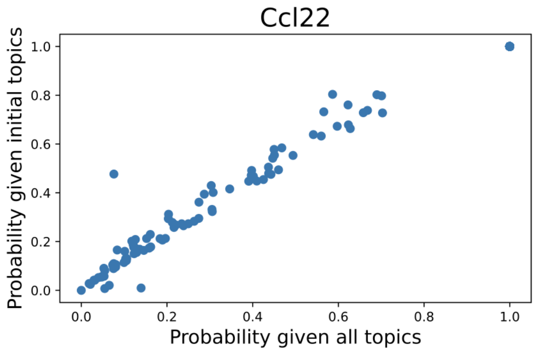

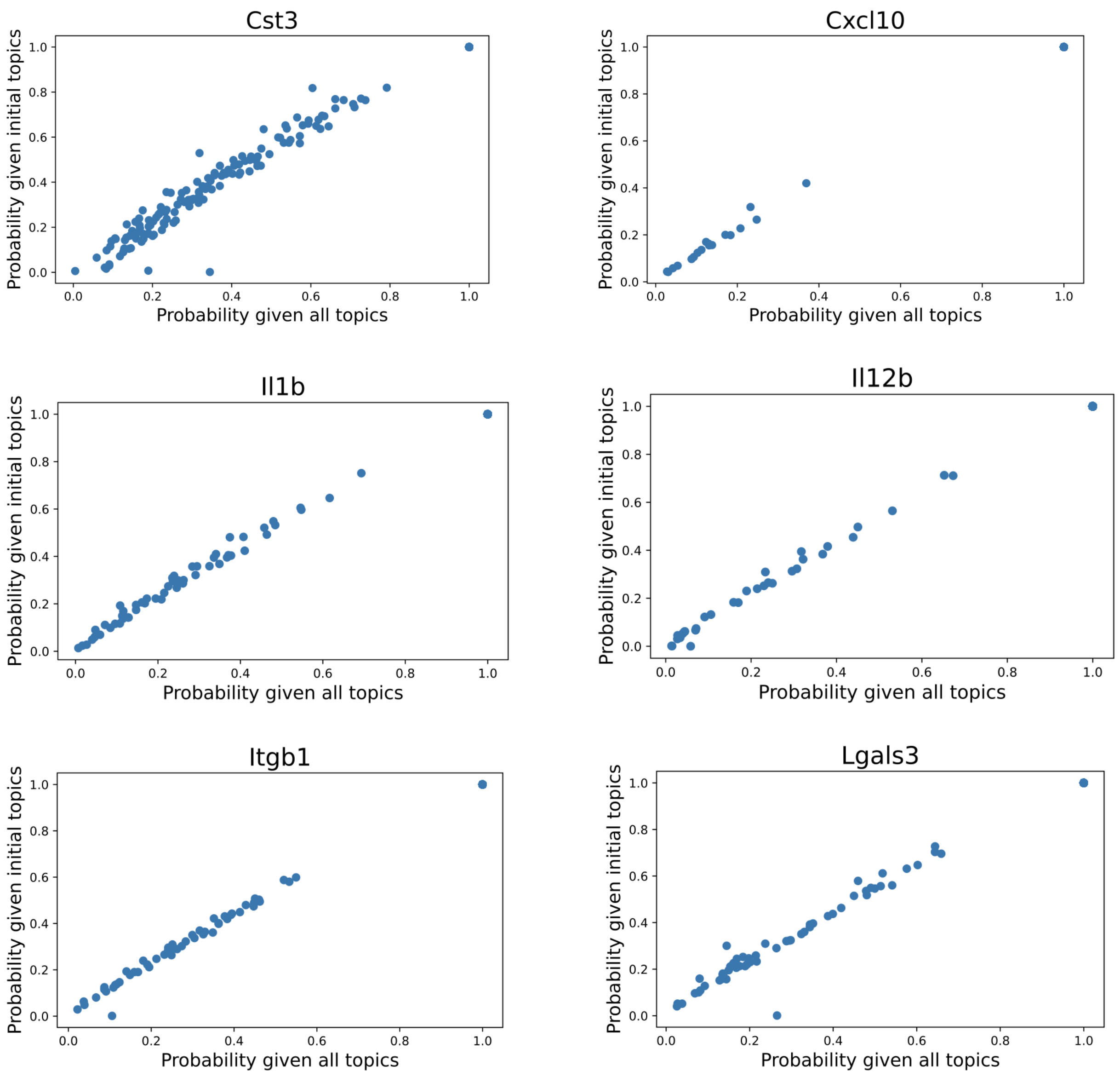

Overall, here we show that the way new topics are used is independent of what genes are being modified in this synthetic experiment. As results were similar to our original experiments we did not discuss this further in the manuscript. Just added the following text: “A further experiment was performed where we upregulated a random set of genes Gcfc2, Wdsub1, AU040320, Pank3, Dcaf12, Gm26669, Ehd2, Bag3, Rpl10-ps2, Notch1, Ppm1g, Oxsr1, Nrarp, Ppp3ca, Rpl28-ps1, Stbd1, Srgap2, Cpped1, Gm10420, St6galnac3. Results can be seen in the supplementary S4 Fig and S5 Fig”,

4. Fig 3 please add total # genes predicted to the legend so the number of false positives can be understood in context.

We have added the following line to the caption: “Total list of ranked genes for those datasets is over 2500”.

5. For the PIC-seq data, thanks for the additional plots, they help with the analysis. A couple questions do arise. Why don’t the cells cluster by time point within type (T cell or DC) in Figs 4, S6? More importantly, I am still left wondering about the number of topics used here. In total, about 1/3 of the PIC-seq topics have been described by the authors, which is indeed helpful although some of them are not informative about immune processes as they contain mostly housekeeping genes. What are the remaining 2/3 of the topics characterizing? I would not hammer on this point so much were it not (as I understand it) the essential core of the methods. Thus I would like to understand if the other topics represent signal (if so what processes?) or background? If the latter, the method may still be useful but there needs to be some way to understand/characterize/rank(?) The topics.

We apologise for the confusion caused by Fig. 4 and S6. We have revised the original topic figure to now include annotations of the timepoints. Thus, S6 is redundant and has been removed. In the revised new figure showing topics (Fig.5) we are now showing the results for 10 topics. As it can be seen from the figure, there are topics associated with cell type and timepoint. Specifically, topics 0 and 1 are DC specific but topic 0 is higher at 20h while topic 1 is higher at 3h. Additionally, topic 5 is T-cell specific for the 48h. Topic 6 is also T-cell specific but for the 3h timepoint. Full list of topics interpretations for Step 1 LDA with 10 topics is available below and also as updated supplementary table 2.

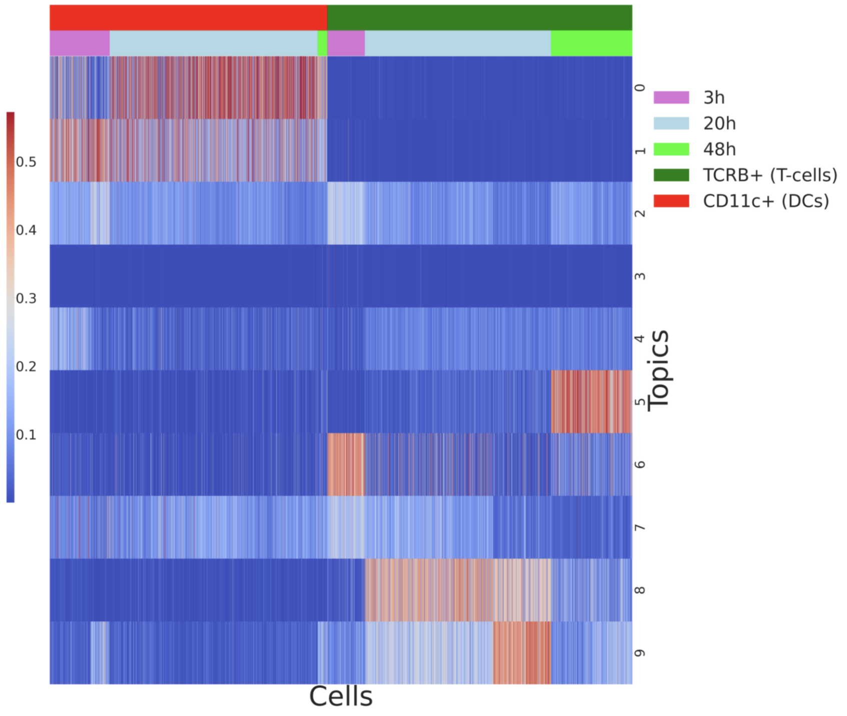

This figure shows the topic expression across the reference population. Those are the results from step 1 of our analysis. This LDA we fit on what we call a reference population; this could be population before interaction has occurred or in the case of the PIC-seq data co-cultures of the single cell types, one co-culture for DCs and one for T-cells. It is expected when fitting LDA on single cell dataset to discover cell type specific topics, general topics, or even topics dominated by mitochondrial and ribosomal genes giving information about the quality of the sample. The topics of the first LDA we do not rank as we fix all of them before proceeding to step 2 of our approach and the analysis of the interacting population. However, we understand the importance of knowing what topics capture and below can be found a full list of the topics, genes with high probabilities in those topics and an explanation of what those topics are. S2 Table has been updated as seen below as we are currently using 10 topics for the Step 1 LDA.

Topic ID	Genes	Notes
0	Fscn1, Calm1, Tmem123, Cd74, Malat1, Ftl1	Generally high expression in DCs, particularly 20h and 48h
1	Cst3, Ccl5, Cd74	DC specific genes, higher in 3h and some 20h
2	mt-Rnr2, mt-Rnr1, mt-Cytb, mt-Nd4, mt-Nd1	Mitochondrial genes; not specific to a cell type topic
3	Igkc, Ighm, Igha, Gm42418, Gm26917, Jchain, Iglj1, B2m	Not specific to a cell type.
4	Cdkna1a, Hspa5, Nfkbia, Ubc, Esd, Nr4a3	Similar expression across but some slightly higher in some T-cells
5	Snord32a, Ldha, Npm1, Ly6a, Eef2, Eef1a1, Trac	High in 48h T-cells
6	Ly6e, Trac, Stat1, Cd52, Gbp2,	High in 3h T-cells
7	Gm42418, Gm26917, Hsp90ab1, Actb, Calr, Lars2, Myh9	Similar expression but slightly lower in some T-cells
8	Npm1, Ncl, Ldha, Ddx21, Nop58, Ccnd2,	High in 20h T-cells
9	AC117232.5, Snord32a, Snord15b, Gm15710, Gm9794, Gm13456, Snord55, Rack1, Mir3091	High in 20h T-cells subset

(New) S2 Table: Genes with high probabilities appearing in the topics identified in the reference

The initial query was regarding our initial analysis. For the current number of topics 10 as seen from the table above, we have provided interpretation of all topics. For completeness, we added further interpretation to more topics from the first LDA with 30 topics.

Topic ID	Genes	Notes
0	Ccr7, Ftl1,Tmsb4x, Id2, Dusp5, Nr4a3, Serpinb9,Cd83,Id2	Genes with higher expression in DCs (mostly 3h)
1	Cd74, Cst3, Ccl22, Ftl1, Malat1	DC higher genes
2	Il2, Ccl4, Ccl3,	Some immune genes but not high expression across celltypes
3	Igkc, Ighm, Igha, Gm26917, Jchain, B2m, Iglj1, Ftl1, mt-Rnr1, Gm15344	Genes not generally expressed in T-cells and DCs. Also housekeeping genes
4	mt-Nd1, mt-Nd2,mt-Nd5, Hnrnpa2b1, Gm28437,'Slc25a3	Mitochondrial
5	Malat1, Cst3, Ccl5, Calm1, Ccl22, Ccr7	DCs 20h
6	Psme2, Psmb8, S100a11, Stat1, Gbp2	DCs 20h and some 48h
7	Snord55, Snora69, Hspe1, Hspa8, Cox7b, Atp5j, Atp5g3	Higher expression in some T-cells
8	Similar to above	Similar to above
9	Mif, Snord32a, Cdkna1a, Eef2	Slightly higher in some T-cells
10	Eef1a1, Snord15b, Snord32a, AC117232.5, Snord35b, Mir3091, Gm13456, Rack1, Gm9794, Gm27390, Gm15710, Snord73, Gm10076, Snord55, Wdr89	Genes with higher expression in T-cells
11	Cd69, Ccnd, Nr4a1, Cfl1, Trac, Hspa5, Nfkbia, Myc, Dusp2, Irf4, Nfkbid, Ly6a, Lcp1, Eif1, Snord14d, Cd40lg, H2-K1, Tnf	Includes genes with higher expression in subsets of T-cells
12	Calm1, Fscn1, Ccl22, Tmem124, Malat1	DC genes high expression
13	Hspa8, Txn1, Psma7, Psme1, Snord14d	Slightly higher 20h T-cells
14	B2m, Actb, Gzmb, Gzma, Il2ra	Similar expression in T-cells an DCs.
17	Gbp2, Stat1, Ly6a,	T-cells 3h and some T-cells 20h
21	Trac, Ly6e, Cd52, Cd53, Cd3d	T-cells 3h, some 20h and some 48h

22	Cfl1, Snord15b, Ldha, Snord32a, Mif	Slightly higher in T-cells
23	Ncl, Snora75, Trac, Tmsb4x, Mif, Rack1, Nop58	T-cells higher expression
25	Ldha, Npm1, Mif, Top2a, Ly6a, Snord32a, Eef2, Eef1a1	Genes with higher expression in subset of T-cells (48h timepoint)
26	Ccl5, Cd74, Ftl1, Bcl2a1b, Il4i1, Il12b, Ifit3m, H2-Ab	Genes with higher expression in subset of DCs (3h timepoint)
27	Gas5, Rac2, Rack1, Coro1a	Slightly higher in T-cells
28	Mitochondrial and housekeeping genes	Similar to 29
29	mt-Nd4, mt-Nd2, AC117232.5, Gm10076, mt-Nd1, B2m, mt-Nd5, mt-Co1, mt-Tt, Fth1, Gm9794	Includes mostly mitochondrial and some housekeeping gene

We do recognise the importance of deciding on the number of topics and we hope that our revised experiments as outlined earlier clarify our approach and also provide guidance for choosing the number of topics for future analysis.
